# Hospital and Institutional News

**Published:** 1920-09-04

**Authors:** 


### HOSPITAL AND INSTITUTIONAL NEWS.

### DEFAULTING EMPLOYERS.

If the local employers had only followed their workpeople's example the annual report of the Llanelly Hospital would have provided an object-lesson to the voluntary system. Of the institution's total income last year of £4,727 no less than £3,063 was subscribed by the working men, the employers of labour contributing only £729. It is a great pity, as the Mayor, Mr. Dan Williams, remarked at the annual meeting, that the names of certain works proprietors are still missing from a list which includes handsome subscriptions from their work-people, and that while some employers have faithfully carried out the undertaking arrived at a few years ago to add fifty per cent. to their employees' contribution, others have failed to honour their bond. The town's war memorial will take the form of a large scheme of extension and additional wards for the hospital, and the local ex-soldiers have resolved to devote the whole of the "Byng money" to which they became entitled, namely, some £1,800 or more, towards that object. The extensions will cost about £25,000, so that the employers, who cannot have enjoyed the recent disclosures, have an opportunity here of fulfilling their neglected promises.

### HOSPITAL SITE IN GOLDEN SQUARE.

The freehold site of the Hospital for Diseases of the Throat in Golden Square, Regent Street, is to be sold. The property, which has three parts, and occupies some 7,130 ft., has lately much increased in value, partly because the woollen industry has made a local centre in the district. The sale, then, would enable the committee to move elsewhere. The hospital was established in 1863 and possesses fifty-nine beds.

### THE SMALLER SATURDAY AND SUNDAY FUNDS.

An interesting innovation is proposed to increase the collections of Hospital Saturday in Kenilworth. Hitherto the employees of the town have received collecting sheets from the Hospital Saturday Committee. They have now been asked in lieu of this to arrange for each employee to contribute a penny a week all the year round. Both parties have received the plan with sympathy, and if adopted the scheme will commence at the beginning of September, after the current collection which was held on August 28. In many of the smaller centres the new plan could be adopted. It would benefit not only the fund, but the donors, who would not miss their weekly penny and at the same time be spared the shilling or more which they have given in a lump sum hitherto. Cannot the same plan be adopted for the Sunday funds? It would not interfere with the church collections, but would put upon a proper basis those outside collections which are more and more common. On them the Sunday fund totals now largely rely; and however many local bodies from the schools upwards agreed to the proposed plan there would still be room for street collections and parades from those not subscribing weekly.

### A COMMON-SENSE COMMENT.

In noticing the approaching meeting of the National Association for the Prevention of Tuberculosis, to be held at Liverpool in October, our contemporary the *Glasgow Herald* speaks true words of wisdom on the main subject to be discussed—viz. the efficacy of the methods of prevention and treatment now in vogue. This subject, it says, cannot be practically dealt with apart from the social and economic causes of the disease. If there is a deficency of nourishing food, or if there is no improvement in housing or atmospheric conditions, no measures of treatment or prophylaxis are likely to be much good. A comparison of the figures for overcrowded districts with those for better class suburbs forces upon society the central fact as to the etiology of tuberculosis—namely, that it is a "misery" disease. If these matters are untouched the campaign against tuberculosis will exhaust itself in mere talk. Speaking of the ravages of tuberculosis on the Continent caused by war privation, our contemporary points to Poland as perhaps the worst sufferer of all. . . . "The miserable populace, to whom the respite of peace is denied, is being swept off in thousands by tuberculosis and hunger typhus. That this clamant argument for peace should not have deterred the Polish Government in its insane advance on Kieff . . . is a bitter commentary on the essential inhumaneness of war." We welcome these home truths all the stronger as coming from a lay source. Years ago now, Sir Robert Giffen said one could not contemplate the position of the poor without wishing for something like a revolution. That wish will now not be long in getting realised, if governments and rulers go on ignoring the woe of the people who set them where they are.

### THIS WEEK'S DRUG MARKET.

The position generally is much the same as last week, and buyers are still inclined to hold off. The restricted buying and the fact that German products are coming in more freely obviously lead to the accumulation of stocks; in the circumstances, therefore, a firming up in prices in the class of products concerned does not seem probable. On the contrary, it is more likely that the easier tendency in the prices of synthetic drugs will continue. Phenacetin, phenazone, aspirin, salicylic acid, hexamine, and resorcin are among the products with an easier price tendency. The price of atropine is lower, as is also that of cocaine. Supplies of potassium bromide are accumulating. At the recent public sales of drugs in Mincing Lane unusually large quantities were offered, but comparatively little sold.

